# Modifiable Psychological Factors are Associated With Clusters of Pain, Fatigue, Fecal Incontinence, and Irritable Bowel Syndrome-Type Symptoms in Inflammatory Bowel Disease: A Latent Profile Analysis

**DOI:** 10.1093/ecco-jcc/jjae183

**Published:** 2024-12-05

**Authors:** Vari Wileman, Joseph Chilcot, Christine Norton, Ailsa Hart, Laura Miller, Imogen Stagg, Natasha Seaton, Richard Pollok, Qasim Aziz, Rona Moss-Morris

**Affiliations:** Department of Psychology, Institute of Psychiatry, Psychology and Neuroscience, King’s College London, London, UK; Department of Psychology, Institute of Psychiatry, Psychology and Neuroscience, King’s College London, London, UK; Florence Nightingale Faculty of Nursing, Midwifery and Palliative Care, King’s College London, London, UK; IBD Unit, St Mark’s Hospital, London, UK; Faculty of Medicine, Imperial College, London, UK; Unit for Social and Community Psychiatry, Wolfson Institute of Population Health, Queen Mary University of London, London, UK; IBD Unit, St Mark’s Hospital, London, UK; Department of Psychology, Institute of Psychiatry, Psychology and Neuroscience, King’s College London, London, UK; Department of Gastroenterology, St George’s University Hospitals NHS Foundation Trust, London, UK; Institute for Infection and Immunity, St George’s University of London, London, UK; Wingate Institute of Neurogastroenterology, Queen Mary University of London, London, UK; Department of Psychology, Institute of Psychiatry, Psychology and Neuroscience, King’s College London, London, UK

**Keywords:** Symptom profiles, psychological, cognitive behavioral

## Abstract

**Background:**

Inflammatory bowel disease (IBD) causes fatigue, pain, and fecal urgency/incontinence symptoms. Identifying symptom profile subgroups and related psychological correlates might enable earlier intervention and more effective tailored treatment pathways.

**Methods:**

This study was nested within a randomized controlled trial of a digital symptom intervention for people with IBD (*n* = 780). Latent profile analysis was conducted on pre-randomization baseline measures of fatigue, pain, and fecal incontinence. Multinominal logistic regression examined associations between profile membership and clinical, demographic and psychological factors.

**Results:**

Latent profile analysis determined a three-profile model: Moderate (50%), High (40%), and Severe symptoms (10%). Diagnosis and fecal calprotectin were not associated with profile membership, but female gender, comorbidity, time since diagnosis, and irritable bowel syndrome (IBS)-type symptoms were associated with High and Severe symptoms profiles. Depression, anxiety, negative symptom perceptions, all-or-nothing and avoidance behaviors significantly increased the relative risk of High and Severe symptoms profile membership.

**Conclusions:**

Many participants experienced symptoms even when deemed to be in clinical remission. After controlling for clinical, inflammatory, and demographic factors, the relative risk of High or Severe symptom profile membership was associated with potentially modifiable cognitive behavioral factors. These factors were also associated with IBS-type symptoms. Recognizing the potential impact of cognitive behavioral factors in exacerbating symptoms can lead to earlier identification of patients who require support and allows treatment plans to be tailored more precisely. The findings from this study promote a more integrated approach to IBD management, combining medical treatment with cognitive behavioral interventions to enhance patient care and improve outcomes.

## 1. Background

Inflammatory bowel disease (IBD), which includes Crohn’s disease and ulcerative colitis (UC), is a long-term, relapsing-remitting gastrointestinal condition affecting over 6 million people worldwide, with increasing prevalence.^1^ The pathophysiology of IBD involves complex genetic, environmental, microbial, and immune factors.^2^ Medications are used to control inflammation and alleviate symptoms. Surgical removal of diseased portions of the gastrointestinal tract is sometimes necessary to manage disease processes.

Pain, fatigue, and fecal urgency/incontinence are commonly experienced IBD symptoms. In a recent UK survey of people with IBD (*n* = 8486), 42% reported the need for help with their pain, 56% for fatigue, and 53% for fecal incontinence.^3^ Many people with IBD continue to experience these and other debilitating symptoms when inflammation is absent or relatively mild, which highlights the need for a broader explanatory model of symptom perpetuation beyond inflammatory processes. Persistent symptoms without active disease inflammation might be understood through the ‘gut–brain axis’, whereby a bidirectional communication network of signaling pathways between the nervous system, immune and hypothalamic–pituitary–adrenal axis, and gastrointestinal tract link the emotional and cognitive centers of the brain with intestinal functions. Any dysregulation of this network, often influenced by factors such as stress and anxiety, may play an important role in maintaining symptoms.^2^ This might also explain why approximately one-third of patients with IBD report symptoms compatible with irritable bowel syndromes (IBS), such as abdominal pain, diarrhea, and bloating.^4^ People with IBD who report IBS-type symptoms also tend to have higher scores for depression, anxiety, and psychological distress compared to those who do not report such symptoms.^5^

The cognitive behavioral model of symptom perpetuation highlights a broader range of psychosocial factors that may contribute to the brain–gut signaling in IBD, exacerbating the symptom experience.^6^The model recognizes that while disease inflammation initiates symptoms, it is often the interaction of cognitive, emotional, and behavioral factors that affects the perception and experience of symptoms. For example, believing that pain and fatigue indicate damage to the body can lead to heightened awareness, distress, and anxiety. In response, individuals may engage in avoidance behaviors, such as reducing physical activity or social interactions, to prevent exacerbation of symptoms. This reduced physical activity and distress affects physiological changes, which can worsen and maintain existing symptoms. By targeting cognitive, emotional, and behavioral factors that may perpetuate persistent symptoms in individuals with IBD, interventions may help alleviate symptom burden, improve quality of life, and promote overall well-being.

The IBD-BOOST intervention, based on a cognitive behavioral model of symptom perpetuation, was recently developed to help people manage their IBD symptoms of fatigue, pain, and fecal urgency/incontinence.^7^ There is a need for this integrated approach; 29% of the UK IBD Survey respondents wanted help for all 3 symptoms of fatigue, pain, and fecal incontinence.^3^ However, it is less well known how these symptoms cluster in individuals; do people experience all 3 symptoms to the same degree or are their subgroups who predominantly experience 1 or 2 of these? Previous symptom profile studies have looked broadly at how physical and psychological symptoms cluster but not specifically fatigue, pain, and fecal incontinence.^8–11^ Psychological symptoms in these studies focused on emotional factors (anxiety and depression) which are important as over 30% of people with IBD experience anxiety and 25% experience depression.^12^ However, in a more recent IBD profile study, illness perceptions (a cognitive factor) were found to have a predictive relationship with symptom cluster trajectories.^13^

The IBD-BOOST intervention targets cognitive (negative symptom perceptions and low self-efficacy for managing the illness) and behavioral factors (all-or-nothing behavior patterns and avoidance of activity in response to symptoms), known to relate to distress, quality of life, and individual symptom severity in IBD^14–18^ as well as emotional factors (depression and anxiety). By addressing these interrelated aspects of psychological functioning, the intervention aims to enhance participants’ quality of life and their symptom experience.

**Figure 1:**
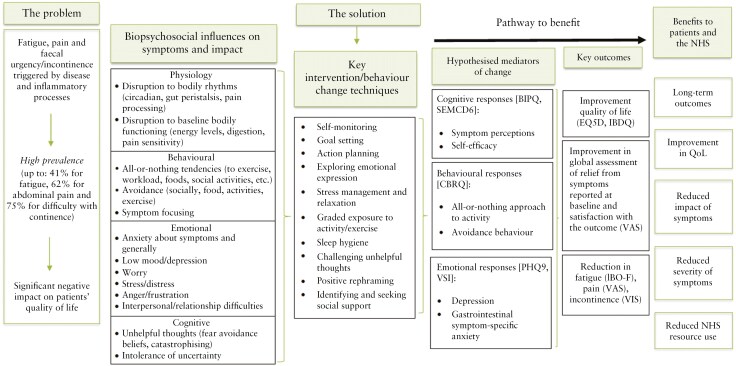
The IBD-BOOST intervention logic model – Published (and can be reproduced) under the terms of Creative Commons Attribution 4.0 Licence) from Sweeney et al Page 7.^7^

The purpose of this study was to examine the relationships between the proposed mechanisms of change in the intervention logic model (Figure 1) and symptom clusters in IBD. Specifically, we aimed to (1) explore whether there are distinct subgroups in the trial participant sample, with symptom profiles based on reporting of pain, fatigue, and urgency/incontinence and (2) examine to what extent the emotional, cognitive, and behavioral response variables, targeted in the intervention, were associated with identified symptom profile groups, and (3) examine the relationships between IBS diagnosis and both the symptom profiles and the emotional, cognitive, and behavioral factors. This approach can provide insights into the heterogeneity of experiences among people with IBD and may lead to more personalized treatment approaches and interventions.

## 2. Methods

### 2.1. Study design and setting

This is a cross-sectional study of baseline data collected as part of a UK National Institute of Health Research (RP-PG-0216-20001) randomized controlled trial: a supported online self-management intervention for symptoms of fatigue, pain, and urgency/incontinence in people with IBD: the IBD-BOOST trial [Trial registration: https://doi.org/10.1186/ISRCTN71618461].^14^ Favorable ethical opinion was granted by the NRES Research Ethics Committee & Health Research Authority (London—Surrey Research Ethics Committee/19/LO/0750). The data that support the findings of this study are available from the corresponding author on reasonable request subject to appropriate data sharing permissions.

### 2.2. Participants and procedure

Participants were recruited from a UK national survey sent by post or an electronic link in an email to unselected patients with IBD from IBD clinic hospital sites, the UK national IBD BioResource, or the charity Crohn’s & Colitis UK.^3^ Eligible participants consenting to participate in further research were invited to the IBD-BOOST randomized controlled trial and completed online consent and trial baseline questionnaires prior to randomization.

### 2.3. Inclusion/exclusion criteria

Eligible patients were aged 18 years old or over and living in the UK (England, Scotland, or Wales), had a diagnosis of IBD (self-reported as having been medically diagnosed with IBD, including patients with an ileo-anal pouch or stoma), and self-scored one or more symptoms of fatigue, pain, or urgency/incontinence as having an impact on their quality of life as 5 or more on a 0-10 scale.^3^ All participants were required to understand written English, be able to give informed consent, and be able to access the online intervention via a computer or mobile device. Patients were excluded if they had one or more ‘red flags’ (such as new bleeding, rapid weight loss, or vomiting, which had not been previously reported to a health care practitioner), which were not currently under adequate investigation and management.

### 2.4. Measures

#### 2.4.1. Symptoms

Pain was measured with the Pain Intensity Numerical Rating Scale (NRS), with 4 items measuring current pain intensity, as well as lowest, worst, and average pain intensity over the last 7 days. Each item was evaluated using an 11-point (0-10) Likert scale. A composite average score was calculated by averaging the 4 items to indicate a pain severity index (0-40).^16^ Fatigue was assessed with the IBD-Fatigue (IBD-F) scale. Items are scored on a 5-point (0-4) Likert scale, with a total score ranging from 0 to 16. A score of 0 indicates no fatigue. Higher scores indicate higher levels of fatigue.^17^ Fecal incontinence was measured with the Vaizey incontinence measure, a 7-item scale that assesses the severity of fecal incontinence by evaluating aspects of bowel control including frequency, type of incontinence, and lifestyle impact. Items are scored on a scale from 0 to 4 or from 0 to 2, with higher scores indicating greater severity or frequency of symptoms. The total score ranges from 0 (perfect continence) to 24 (total incontinence) and is obtained by summing individual item scores to provide an overall measure of fecal incontinence severity [continuous].^18^

#### 2.4.2. Disease activity

Fecal calprotectin was measured through analysis of stool samples, as an objective indicator of intestinal inflammation and disease activity. A cutoff of 200 μg/g was treated as an indicator of disease inflammation.^19^

### 2.5. Comorbidity

Medically diagnosed physical health conditions (heart, cancer, thyroid, kidney, diabetes, respiratory including asthma, liver disease, major neurological, or nerve problems (such as multiple sclerosis) or any other major illness or disease) and medically diagnosed mental health conditions (anxiety, depression treated by medication, therapy or counseling in the past year, and other mental health illness (eg schizophrenia or bipolar disorder) were recorded and a total comorbidity score was calculated.

### 2.6. IBS-type symptoms

The presence of IBS-type symptoms was measured to investigate whether trial participants with IBS-type symptoms at baseline responded differently to the IBD-BOOST intervention. The Rome IV diagnostic criteria include recurrent abdominal pain on average at least 1 day/week in the last 3 months, associated with 2 or more of the following criteria: related to defecation, associated with a change in frequency of stool or associated with a change in form (appearance) of stool.^20^

### 2.7. Psychological factors

#### 2.7.1. Emotional response measures

The Patient Health Questionnaire-9 (PHQ-9) was used to measure depression symptom severity (19). For each item, responses range from 0 to 3 (ie not at all [0]; several days^1^; more than half the days^2^; nearly every day^4^). The total score ranges from 0 to 27. A score of 0-4 indicates no depression, 5-9 indicates mild depression, 10-14 indicates moderate depression, 15-19 indicates moderately severe depression, and 20-27 indicates severe depression.^21^

The Visceral Sensitivity Index was used to gastrointestinal symptom-specific anxiety using 15 questionnaire items, with responses ranging from 1 (ie strongly agree) to 6 (ie strongly disagree). Items are scored on a reversed 6-point scale ranging from 0 to 5, with sum scores between 0 and 75. Higher scores indicate more severe symptom-specific anxiety.^22^

#### 2.7.2. Cognitive response measures

The Self-Efficacy of Managing Chronic Diseases Scale (SEMCD) was used to measure how confident participants were in completing certain activities relevant to their condition. The measure consists of 6 items that are rated on a 10-point scale ranging from ‘not at all confident’ (1) to ‘totally confident’ (10). A mean was calculated, where higher scores indicate greater self-efficacy for managing the chronic condition.^23^

The Brief Illness Perceptions Questionnaire (BIPQ) was used to measure IBD illness-specific symptom perceptions. It is an 8-item scale, with items rated on an 11-point Likert scale. Each item assesses one dimension of symptom perceptions, including serious consequences, timeline (chronic), personal control, treatment control, identity, coherence, emotional representation, and symptom concern. A composite BIPQ score was computed with the individual 8 domain scores summed together (personal control, treatment control, and coherence items were reverse scored). A higher BIPQ score indicates a more negative overall perception of symptoms, that is, which symptoms have serious impact, are ongoing, confusing, and uncontrollable.^24^

#### 2.7.3. Behavioral response measures

Two subscales of the Cognitive and Behavioural Responses to Symptoms Questionnaire (CBRQ) were used to measure patients’ behavioral responses to symptoms. (1) All-or-nothing behavior (5-25), for example, I tend to overdo things and then rest up for a while and (2) Avoidance/resting behavior (8-40), for example, When I experience symptoms, I rest. Higher scores indicate higher All-or-nothing or Avoidance/resting behavior.^25^

### 2.8. Statistical analysis

Latent profile analysis (LPA) is a statistical modeling technique to identify subgroups or “profiles” within a heterogeneous population into discrete groups or latent classes, based on participants’ responses to multiple continuous variables. The statistical analysis method identifies the smallest number of latent groups required to best represent the patterns in the variables’ data.^26,27^ Latent profile analysis was conducted on pre-randomization baseline measures of fatigue (IBD-F), pain (NRS), and fecal incontinence (Vaizey). As they could not contribute to the data on fecal incontinence, participants with a stoma were excluded from analysis. We followed a common applied LPA modeling procedure.^28^ To determine the best-fitting symptom profile, clinical interpretability of the model and sample size of each profile were considered alongside standard model fit statistics, including, the log-likelihood (LL), Akaike Information Criterion (AIC), Bayesian Information Criterion (BIC), Vuong Lo-Mendell-Rubin likelihood ratio test (VLMR-LRT), Lo-Mendell-Rubin likelihood ratio test (LMR-LRT), and Parametric bootstrapped LL ratio test, and model entropy. We tested 1-5 profile models. Each model was rerun, doubling the number of random starts in Mplus to check that the best model LL was replicated. Although the fourth and fifth profiles showed a modest improvement in model entropy and significant improvement in 1 of the *k-*1 tests (parametric bootstrapped LL ratio test), the sample sizes were small with only 19 and 9 people, respectively. After the most appropriate profile solution was determined, multinominal logistic regression, with inverse probability sample weights, examined associations between profile membership (dependant variable) and clinical, including inflammatory (fecal calprotectin), demographic, and psychological factors also collected at baseline. Correlations between independent symptoms and psychological variables were also conducted. Analyses were conducted in Stata v17 and Mplus v8.

## 3. Results

**Figure 2:**
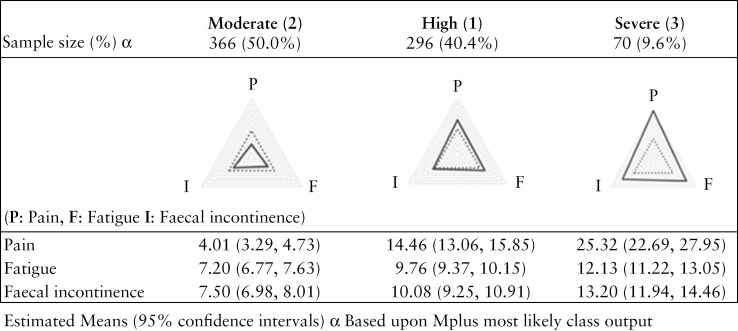
Symptom radar plots and estimated symptom means of the three latent profile groups.

Seven hundred and eighty participants consented to participate in the trial and completed baseline questionnaires. Forty-eight participants with a stoma were excluded from the analysis as data on incontinence was not relevant to them. The analysis sample of 732 participants had a mean age of 49 years, 493 (67%) were women, 391 (53%) had Crohn’s disease (CD), and 389 (47%) UC. Five hundred and sixty-three (77%) participants provided a fecal sample, and fecal calprotectin analysis indicated intestinal inflammation (≥200 μg/g) for 106 (19%); therefore, 81% of the trial participants, who provided fecal calprotectin samples, were considered to be in clinical remission. The median disease duration was 11 years (IQR-5-23). Three hundred and forty-eight (47%) participants met the Rome IV criteria for IBS, of which 267 (77% were women). In the 81% of participants considered to be in clinical remission, those meeting the Rome IV criteria increased to 50%. Symptom means were fatigue (8.7, SD = 3.5), pain (10.3, SD = 8.0), and fecal incontinence (9.1, SD = 5.0) (Figure 2). A correlation matrix is included in the Supplementary Appendix 2 reporting univariate analyses between independent pain, fatigue, and fecal incontinence measures and psychological variables.

### 3.1. Latent profiles

A 3-profile model (first *n* = 296, second *n* = 366, and third *n* = 70) had the most appropriate fit and interpretation (see Supplementary Appendix 1). The 3-profile solution consisted of: Moderate (2) symptoms profile (*n* = 366, 50%), High (1) symptoms profile (*n* = 296, 40%), and Severe (3) symptoms profile (*n* = 70, 10%). Radar Z-score plots demonstrate that all 3 symptoms (pain, fatigue, and fecal incontinence) were accounted for in each profile with varying levels of severity; supported by estimated model means shown in Figure 2.

### 3.2. Multinominal logistic regression—demographic and clinical factors

Diagnosis (CD or UC) and fecal calprotectin were not associated with profile membership, but female gender, multimorbidity, time since diagnosis and the presence of Rome IV criteria for the diagnosis of IBS were associated with High and Severe symptoms profile membership previous IBD surgery was associated with Severe symptoms profile membership only (Table 1).

**Table 1:** Demographic, clinical, and psychological factors relative risk associated with latent profile groups.

	Unadjusted RR (95% CI)			
Demograhic and clinical variables	Profile High (1)	Profile Severe (3)		
Age	0.99 (0.98, 1.00)	0.98 (0.96, 1.00)		
Gender	0.60 (0.44, 0.84)**	0.48 (0.24, 0.94)*		
Combined comorbidity total	1.41 (1.21, 1.63)**	1.82 (1.49, 2.24)**		
Diagnosis (CD vs UC/other IBD)	1.08 (0.79, 1.47)	0.62 (0.36, 1.06)		
Time since diagnosis	0.98 (0.97, 0.99)*	0.97 (0.92, 0.99)**		
IBD surgery α	0.94 (0.67, 1.33)	1.79 (1.04, 3.08)*		
Rome classification α	4.54 (3.23, 6.37)**	8.11 (4.38, 15.01)**		
Calprotectin ≥200	1.29 (0.82, 2.03)	1.34 (0.64, 2.83)		
α yes vs no				

	Unadjusted RR (95% CI)		Adjusted RR (95% CI)^a^	
Psychological variables	Profile High (1)	Profile Severe (3)	Profile High (1)	Profile Severe (3)
Depression	1.17 (1.13, 1.22)**	1.42 (1.34, 1.51)**	1.15 (1.11, 1.20)**	1.43 (1.33, 1.53)**
Anxiety	1.05 (1.03, 1.06)**	1.12 (1.09, 1.15)**	1.04 (1.02, 1.05)**	1.11 (1.08, 1.14)**
Self-efficacy	0.68 (0.61, 0.75)**	0.41 (0.35, 0.49)**	0.74 (0.66, 0.82)**	0.45 (0.37, 0.54)**
Symptom perceptions	1.06 (1.04, 1.08)**	1.16 (1.13, 1.20)**	1.05 (1.03, 1.07)**	1.15 (1.11, 1.18)**
All-or nothing behavior	1.10 (1.07, 1.14)**	1.22 (1.16, 1.30)**	1.08 (1.03, 1.12)**	1.19 (1.12, 1.27)**
Avoidance	1.08 (1.05, 1.11)**	1.17 (1.13, 1.22)**	1.07 (1.03, 1.11)**	1.15 (1.09, 1.20)**

^a^adjusted for: gender, comorbidity total, time since diagnosis, IBD surgery, and Rome classification.

Base comparison Profile Moderate (2). Models included inverse probability sample weights *p < 0.01 **p < 0.05. CI, confidence interval; IBD, inflammatory bowel disease; RR, relative risk.

### 3.3. Multinominal logistic regression—psychological factors

All psychological factors measured in this study significantly correlated with all 3 (pain, fatigue, and fecal incontinence) individual symptoms (see Supplementary Appendix 2.) Controlling for demographic and clinical factors associated with profile membership, all psychological measures assessed at baseline and targeted in the IBD-BOOST intervention were associated with profile membership (Table 1). Compared to the Moderate symptoms profile, depression, anxiety, negative illness perceptions, all-or-nothing behaviors, and avoidance all significantly increased the relative risk of being in the High and Severe symptoms profiles. Self-efficacy was associated with a reduction in risk. Those who met Rome IV symptom criteria for IBS had 4.5 times the risk of being in the high symptom profile and 8 times the risk of being in the severe symptom profile group. Those with IBS symptoms also scored significantly higher on all the psychological variables. Profile characteristics for symptom profile groups and Rome IV IBS criteria groups are reported in Table 2 and additional treatment characteristics are in Supplementary Appendix 3.

**Table 2:** Demographic, clinical, and psychological characteristics of latent profile groups and Rome IV criteria for irritable bowel syndrome-type symptom groups.

		Latent profile group							IBS-type symptoms in IBD Rome IV criteria			
	All		Moderate		High		Severe		Criteria not met		Criteria met	
Number of participants	732^a^		366		296		70		384		348	
***Demographic variables***												
Age	48.9	14.3	50.0	14.6	48.1	13.8	46.4	15.0	50.3	14.6	47.3	13.9
Sex, F **(*n*/%)**	493	67.4	221	60.1	217	73.3	55	78.6	226	58.9	267	76.7
Diagnosis Crohn’s **(*n*/%)**	391	53.4	194	53.0	152	51.4	45	65.3	198	51.8	191	54.9
Time since diagnosis (years)	15.0	12.4	16.2	12.7	14.3	12.3	11.6	11.0	16.1	13.0	13.9	11.7
***Clinical variables***												
Pain (0-40)	10.3	*8.0*	3.7	*3.2*	14.6	*3.6*	25.9	*4.3*	6.9	7.2	14.0	7.2
Fatigue (0-16)	8.7	*3.5*	7.1	*3.4*	9.8	*2.6*	12.5	*2.3*	7.9	3.6	9.6	3.1
Faecal incontinence (0-24)	9.1	*5.0*	7.5	*4.7*	10.0	*4.7*	13.5	*4.7*	8.0	4.9	10.2	5.0
Fecal calprotectin (*n* = 563)	170.0	*371.2*	149.7	*366.5*	186.6	*364.9*	220.6	*423.9*	163.7	392.8	176.9	346.9
Rome IV class membership **(*n*/%)**	348	*46.5*	99	*27.0*	195	*65.9*	54	*77.1*	384	0	348	100
***Psychological variables***												
Depression (0-27)	9.1	*5.6*	6.7	*4.4*	10.4	*5.0*	16.5	*6.0*	7.6	5.0	10.8	5.8
Anxiety (0-75)	39.0	*16.9*	32.0	*15.4*	43.6	*14.9*	56.6	*12.8*	32.9	15.7	45.8	15.6
Self-efficacy (0-10)	5.9	*2.0*	6.7	*1.8*	5.3	*1.8*	3.7	*1.6*	6.5	1.9	5.2	1.9
Symptom perceptions (0-80)	42.5	*11.8*	38.1	*11.1*	45.3	*10.2*	54.1	*9.9*	39.1	12.0	46.3	10.3
All-or nothing behavior (5-25)	13.2	*4.9*	11.8	*4.5*	14.1	*4.7*	16.6	*5.1*	12.2	4.6	14.3	5.0
Avoidance/resting behavior (8-40)	18.5	*5.7*	17.0	*5.2*	19.3	*5.8*	22.5	*5.2*	17.5	5.5	19.5	5.8

Data reported: means and standard deviation unless otherwise indicated **(*n*/%)**. Pain (Numerical Rating Scale), Fatigue (IBD-Fatigue Scale), Fecal incontinence (Vaizey incontinence measure), Depression (PHQ-9), Anxiety (Visceral Sensitivity Index), Self-efficacy (SEMCD Scale) Symptom Perceptions (B-IPQ), All or nothing behavior and Avoidance/Resting behavior (CBRQ).

^a^Patients with stoma excluded (*n* = 48).

## 4. Discussion

**Figure 3:**
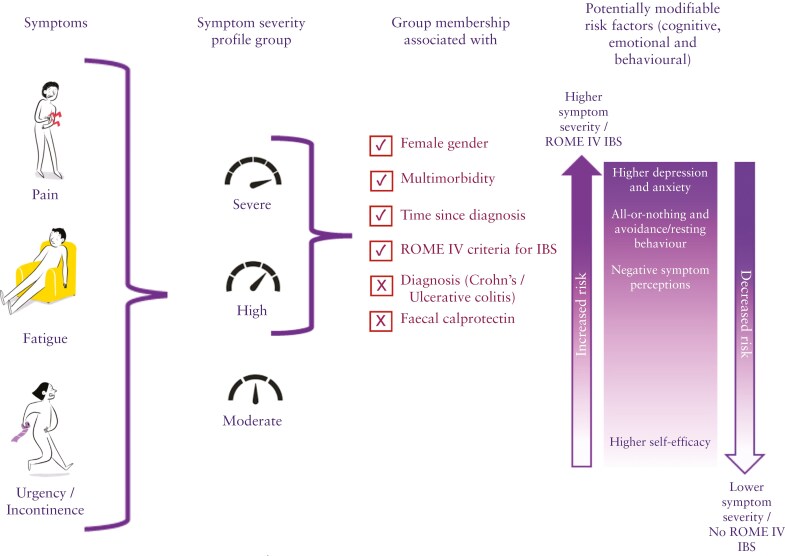
Associated risk factors for symptom severity profile group.

This study identified symptom profile groups in people diagnosed with IBD where the increased presence of potentially modifiable cognitive behavioral factors was associated with the severity of overall symptom experience. Figure 3 presents a descriptive model of the findings of the LPA of IBS symptoms and the associated risk factors.

Latent profile analysis revealed 3 distinct symptom profiles, where all 3 symptoms of interest (pain, fatigue, and fecal incontinence) were accounted for in each profile, but profiles varied in levels of severity. This finding aligns with the logic model developed for IBD-BOOST which, informed by previous research,^14^ theorized that individuals with IBD often experience a combination of symptoms.^7^ Half of the participants in our sample were categorized into the High or Severe symptom profiles, but disease activity indicated by fecal calprotectin, an established marker for gastrointestinal inflammation,^29^ was not associated with profile membership. Many participants experienced symptoms despite having low or no objective inflammation as measured by fecal calprotectin <200 microg/g stool. The high completion rate (77%) of fecal calprotectin samples enhances confidence in this study’s finding that inflammation was not associated with symptom severity and supports findings from previous IBD profile studies.^9,10^

We found no risk of higher symptoms associated with participants’ age or diagnosis (CD or UC) but having multiple conditions in addition to IBD, increased IBD disease duration, and previous surgery were all associated risks with High and Severe symptom profile membership. We also found an increased risk of higher symptoms for women, consistent with previous IBD profile studies; however, in those studies, the profiles included both physical and psychological symptoms and were explained partly by higher depression and anxiety levels reported in women.^8,10^ As men and women are equally affected by IBD, the finding that women in our sample had an increased risk of pain, fatigue, and urgency/incontinence might indicate that their experience of these physical symptoms is complicated by psychological factors.

Those who met Rome IV symptom criteria for IBS were also significantly represented in profile membership. Participants in the High symptom profile were 4 times more likely to report IBS-type symptoms, and those in the Severe profile, 8 times more likely. This is perhaps unsurprising as a meta-analysis estimated 25-35% of people with IBD experience symptoms compatible with IBS whilst in clinical remission^5^ and an IBS diagnosis includes among other symptoms abdominal pain and altered below habit including diarrhea. Fatigue, however, is not included in IBS diagnostic criteria. The important finding from our study is that in addition to higher depression and anxiety levels, people with IBS-type symptoms in IBD also score highly on cognitive behavioral factors related to symptom perpetuation. Rather than viewing these factors as a mental health issue, they highlight how people experience and interpret their symptoms related to illness.

Previous studies have explored IBD symptom profiles based on both physical and psychological symptoms.^8–10^ Our study was nested in a randomized controlled trial for IBD-BOOST, an intervention grounded in cognitive behavioral theory targeting hypothesized psychological mediators related to pain, fatigue, and fecal incontinence symptom experience. Understanding how the psychological factors affected symptom experience at baseline was very important. However, as the intervention was the first to target the 3 physical symptoms of fatigue, pain, and incontinence collectively, it was important for our analysis to explore potential patterns in the symptom burden before examining the associated psychological factors. This study established that after controlling for clinical, inflammatory, and demographic factors, the relative risk of High or Severe symptom profile membership was associated with increased depression, anxiety, symptom perceptions, all-or-nothing and avoidance behaviors, and reduced self-efficacy for managing the illness. Depression, anxiety, and negative symptom perceptions have been associated with increased symptom burden in both IBD and IBS^12,30^ and self-efficacy is related to quality of life.^30,31^ All-or-nothing behavior has been identified as an important factor in IBD fatigue^32^ but this study has now demonstrated that cognitive and emotional behaviors (all-or nothing and avoidance behaviors) are risk factors related to High and Severe symptom profile membership for all three symptoms. All-or-nothing and avoidance behaviors can interact with persistent physical symptoms. For example, an individual with persistent fatigue may engage in excessive activity on days when fatigue improves, causing exhaustion and a prolonged need to rest. Conversely, avoidance behaviors might limit their engagement in activities that could improve overall well-being. Encouraging a more consistent approach to activity and discouraging all-or-nothing behavior can be beneficial.

### 4.1. Strengths and limitations

This study contributes to the existing literature examining profile groups in IBD^8–10^ and is original in examining potentially modifiable emotional and cognitive behavioral factors together. The LPA benefitted from a large sample size, significantly larger than the minimum recommended sample of 500, sufficient for identifying an accurate number of latent profiles.^33^ Obtaining a high percentage of fecal calprotectin data, a known challenge in other IBD profile studies,^8,10^ increased confidence in the interpretation of the study findings after controlling for a clinical marker of active disease. We recognize that the sample consisted of participants recruited to a digital self-management programme who specifically wanted help for pain, fatigue, and/or fecal urgency incontinence symptoms. The sample also had a higher percentage of women than men; it is known that women are more likely to engage in psychological interventions. Participants were also required to speak English and have access to a computer or smartphone. The sample may not be fully representative of the wider IBD population, and this might limit the generalisability of the study findings. As participants were recruited from the UK national IBD BioResource and Crohn’s & Colitis UK charity, as well as National Health Service IBD hospital clinics, we also relied on participants self-reporting IBD medical diagnoses. However, it was reassuring that all participants completed detailed report forms with their medical history and over three-quarters of the sample returned fecal samples for analysis. As the study sample participated in a randomized controlled trial, we were unable to conduct a longitudinal study as in previous reports^10^; our study would therefore benefit from a replication longitudinal study. Finally, we recognize the potential issue of the salsa effect, which refers to the coercion of classes (or profiles) to fit a population that may in fact not have underlying latent classes. While inspection of parameter distributions and parallel plots suggest no significant issues, we cannot rule out the possibility of a partial effect.

### 4.2. Implications for healthcare provision

The identification of distinct symptom profiles provides an understanding of how symptoms cluster together, allowing for more targeted and personalized interventions based on the specific symptom patterns observed in each subgroup. This study shows that there are groups of people with IBD who experience multiple symptoms in clinical remission that are broader than the IBS symptom criteria and interact with psychological factors and persistent physical symptoms. This is interesting because it implies that the presence of IBS-type symptoms and fatigue may not necessarily indicate active disease and the need for escalated medical interventions.^34^ However, the need for support remains and a model of treatment that encompasses psychological approaches needs to be considered.

## 5. Conclusion

Latent profile analysis confirmed that IBD-BOOST trial participants experienced all 3 symptoms of fatigue, pain, and incontinence with varying levels of severity. By highlighting that half of the patients experienced High or Severe symptoms despite the majority being in clinical remission, the study emphasizes the importance of addressing symptoms beyond clinical indicators. This encourages a more integrated approach to patient care that includes both objective clinical measures and subjective patient experiences. Potentially modifiable psychological factors were all associated with symptom experience and independent of objective markers of inflammation. Informing patients about the role of symptom-related/illness-related cognitive and behavioral factors can support them to engage in strategies that may alleviate symptoms. It is, however, worth noting that when psychological factors are broached in relation to symptoms in remission it can leave patients feeling that symptoms are being construed as ‘all in their head’ or a mental health problem. It is, therefore, important to highlight that these are often intuitive responses to symptoms that become less helpful when symptoms move from acute to chronic. Similarly, distress is understandable when symptoms are ongoing and appear intractable. Explaining how these cognitive, behavioral, and emotional factors link to the brain-gut access avoids this duality in thinking. In addition, although inflammation was not related to symptom severity, experiencing multiple conditions in addition to IBD, increased IBD disease duration, and previous surgery highlighted a complex clinical picture. This information is important for understanding the study population and together with the trial analysis, can help guide more personalized and effective approaches to treatment and intervention.

## Supplementary Data

Supplementary data are available online at *ECCO-JCC* online.

jjae183_suppl_Supplementary_File

## Data Availability

The data that support the findings of this study are available from the corresponding author on reasonable request subject to appropriate data sharing permissions.

## Conference Presentation

Society for Biopsychosocial Science and Medicine (formerly American Psychosomatic Society) annual conference UK February 2024.
