# Supplementary material for: Modifiable Psychological Factors are Associated With Clusters of Pain, Fatigue, Fecal Incontinence, and Irritable Bowel Syndrome-Type Symptoms in Inflammatory Bowel Disease: A Latent Profile Analysis
Source: J Crohns Colitis. 2024 Dec 5;19(5):jjae183. doi: 10.1093/ecco-jcc/jjae183 (PMC12087568; doi:10.1093/ecco-jcc/jjae183)
Supplement: jjae183_suppl_Supplementary_File [file jjae183_suppl_supplementary_file.docx]

**Supplementary files**

**Appendix 1 Unconditional Latent Profile Analysis – testing multiple solutions**

|  | Profiles |  |  |  |  |
| --- | --- | --- | --- | --- | --- |
| Parameters | **1** | **2** | **3** | **4** | **5** |
| log-likelihood | -6739.3 | -6612.24 | -6572.58 | -6553.93 | -6539.21 |
| AIC | 13490.6 | 13244.49 | 13173.15 | 13143.86 | 13122.41 |
| BIC | 13518.17 | 13290.45 | 13237.49 | 13226.58 | 13223.52 |
| Entropy | - | 0.7 | 0.78 | 0.8 | 0.82 |
| VLMR-LRT p-value | - | <0.001 | <0.01 | 0.473 | 0.117 |
| Adjusted LMR-LRT p-value | - | <0.001 | <0.01 | 0.48 | 0.121 |
| Parametric Bootstrapped LL ratio test | - | <0.001 | <0.001 | <0.001 | <0.001 |

AIC: Akaike Information Criterion; BIC: Bayesian Information Criterion; VLMR-LRT: Vuong Lo-Mendell-Rubin likelihood ratio test; LMR-LRT: Lo-Mendell-Rubin likelihood ratio test; LL: log-likelihood

**Appendix 2 Univariate analysis IBD symptoms and psychological variables**

|  | P | F | FI | PHQ9 | VSI | SE | BSPQ | AOR | ARB |
| --- | --- | --- | --- | --- | --- | --- | --- | --- | --- |
| Pain [P] |  |  |  |  |  |  |  |  |  |
|  |  |  |  |  |  |  |  |  |  |
| Fatigue [F] | 0.44* |  |  |  |  |  |  |  |  |
|  |  |  |  |  |  |  |  |  |  |
| Faecal Incontinence [FI] | 0.34* | 0.15* |  |  |  |  |  |  |  |
|  |  |  |  |  |  |  |  |  |  |
| Depression [PHQ] | 0.48* | 0.62* | 0.34* |  |  |  |  |  |  |
|  |  |  |  |  |  |  |  |  |  |
| Anxiety [VSI] | 0.49* | 0.41* | 0.43* | 0.58* |  |  |  |  |  |
|  |  |  |  |  |  |  |  |  |  |
| Self-efficacy [SE] | -0.48* | -0.49* | -0.36* | -0.61* | -0.59* |  |  |  |  |
|  |  |  |  |  |  |  |  |  |  |
| Symptom Perceptions [BSPQ] | 0.44* | 0.38* | 0.43* | 0.53* | 0.65* | -0.63* |  |  |  |
|  |  |  |  |  |  |  |  |  |  |
| All or Nothing Behaviour [AOB] | 0.29* | 0.40* | 0.15* | 0.42* | 0.35* | -0.34* | 0.24* |  |  |
|  |  |  |  |  |  |  |  |  |  |
| Avoidance/Resting Behaviour [ARB] | 0.25* | 0.37* | 0.21* | 0.48* | 0.44* | -0.46* | 0.37* | 0.5* |  |
|  |  |  |  |  |  |  |  |  |  |
| *p<0.01, **p<0.05 *Patients with stoma excluded (n=48). Pain (Numerical Rating Scale), Fatigue (IBD-Fatigue Scale) measure) | | | | | | | | | |
| Faecal incontinence (Vaizey incontinence), Depression (PHQ-9), Anxiety (Visceral Sensitivity Index), Self-efficacy (SEMCD scale) | | | | | | | | | |
| Symptom Perceptions (Brief IPQ), All or nothing behaviour and Avoidance/Resting behaviour (CBRQ sub-scales) | | | | | | | | |  |

**Appendix 3 Treatment characteristics of latent profile groups and Rome IV criteria for IBS-type symptom groups**
